# Comparison of Characteristics of Connective Tissue Disease-Associated Interstitial Lung Diseases, Undifferentiated Connective Tissue Disease-Associated Interstitial Lung Diseases, and Idiopathic Pulmonary Fibrosis in Chinese Han Population: A Retrospective Study

**DOI:** 10.1155/2013/121578

**Published:** 2013-09-19

**Authors:** Lin Pan, Yuan Liu, Rongfei Sun, Mingyu Fan, Guixiu Shi

**Affiliations:** ^1^West China School of Medicine, Sichuan University, Chengdu 610000, China; ^2^Department of Rheumatology and Clinical Immunology, The First Hospital of Xiamen University, Xiamen 361003, China

## Abstract

Our study compared the prevalence and characteristics of patients with connective tissue disease-associated interstitial lung disease (CTD-ILD), undifferentiated connective tissue disease-associated interstitial lung disease (UCTD-ILD), or idiopathic pulmonary fibrosis (IPF) between January 2009 and December 2012 in West China Hospital, western China. Patients who met the criteria for ILD were included and were assigned to CTD-ILD, UCTD-ILD, or IPF group when they met the criteria for CTD, UCTD, or IPF, respectively. Clinical characteristics, laboratory tests, and high-resolution CT images were analyzed and compared among three groups. 203 patients were included, and all were Han nationality. CTD-ILD was identified in 31%, UCTD-ILD in 32%, and IPF in 37%. Gender and age differed among groups. Pulmonary symptoms were more common in IPF, while extrapulmonary symptoms were more common in CTD-ILD and UCTD-ILD group. Patients with CTD-ILD had more abnormal antibody tests than those of UCTD-ILD and IPF. Little significance was seen in HRCT images among three groups. A systematic evaluation of symptoms and serologic tests in patients with ILD can identify CTD-ILD, UCTD-ILD, and IPF.

## 1. Introduction 

Interstitial lung disease (ILD) is a heterogeneous group of parenchymal lung disorders that result from variable etiologies but share common radiologic, pathologic, and clinical manifestations [1]. The prevalence of ILD is high and varies from 10.7/100,000 to 27.14/100,000 in different countries [2–5]. Several rheumatologic conditions are associated with the development of ILD [6]. These diseases include systemic sclerosis (SSc), rheumatoid arthritis (RA), polymyositis/dermatomyositis (PM/DM), Sjogren's syndrome, systemic lupus erythematosus (SLE), and mixed connective tissue disease(CTD) [1]. Connective tissue disease-associated ILD (CTD-ILD) refers to patients who are diagnosed as ILD and met the diagnosis criteria for a defined CTD simultaneously. The prevalence of CTD-ILD occupied 19%~34% of ILD [6, 7]. Recently, a large number of ILD patients who have one or several features of systemic autoimmune disease but do not fulfill American College of Rheumatology (ACR) classification criteria for defined CTD have been classified [8]. These patients are considered to have undifferentiated connective tissue disease (UCTD) and take up as many as 25% of ILD patients as reported [8].

Recent studies have shown that CTD-ILD, UCTD-ILD, and IPF were three distinct subgroups of diseases, which differ from prognosis and treatment. Patients with IPF were found to have much worse outcome compared with patients with CTD-ILD and UCTD-ILD [6, 9, 10]. Patients with a diagnosis of CTD-ILD or UCTD-ILD may lead to additional immunosuppressive therapy, whereas a diagnosis of IPF may lead to different therapies other than immunosuppressive therapy to prevent substantial treatment-related side effects. Thus, it is necessary to identify patients of CTD-ILD and UCTD-ILD from IPF. At present, most studies were conducted in USA and European countries, and little knowledge is known in Asia, especially in China. In this study, we retrospectively studied 203 cases of Chinese ILD patients. The prevalence and clinical features of CTD-ILD, UCTD-ILD, and IPF were analyzed. 

## 2. Patients and Methods

### 2.1. Study Population

Patients with a diagnosis of ILD in West China Hospital from January 2009 to December 2012 were selected in this study. ILDs were diagnosed according to the ATS/ERS consensus classification [11]. Patients with environmental exposures and other known causes of ILD were excluded. Patients were classified into three groups (CTD-ILD, UCTD-ILD, and IPF) based on the presence of CTD or UCTD. Study subjects who met the American College of Rheumatology (ACR) criteria for CTD were defined as CTD-ILD group [12–17]. ILD patients who did not meet ACR criteria for connective tissue diseases were defined as UCTD-ILD group if they had at least one sign or symptom suggestive of a connective tissue disease and at least one serologic test supportive of an autoimmune process, as listed in Table 1 [6, 8]. IPF group was defined using the ATS criteria for IPF [11]. Serologic tests were considered positive if the results were above the reference value. Anti-nuclear antibody was considered abnormal only when its titer was higher than 1 : 160. 

### 2.2. Data Collection

Clinical data including detailed patients history, clinical manifestations, laboratory findings, and HRCT findings were obtained from patients' medical records from the first encounter.

### 2.3. Patient History

Clinical manifestations including (1) symptoms related to ILD such as cough, sputum production, and chest distress and (2) symptoms related to CTD such as skin rash, arthralgia, Raynaud's phenomenon, and fever.

### 2.4. HRCT Findings

All high-resolution CT (HRCT) scans were reviewed by two independent doctors from Department of Radiology without knowledge of this study. Signs including consolidation, ground glass opacities, traction bronchiectasis, irregular linear opacities, subpleural curvilinear shadows, and honeycombing were evaluated. 

### 2.5. Statistical Analysis

Patient characteristics, clinical symptoms, HRCT findings, and serologic test results were reported as mean ± SEM or as frequency counts and percentages. The prevalence of clinical findings, serologic tests for antibodies, and radiographic patterns among the three groups was compared using chi-square test or analysis of variance. The serologic tests were calculated using analysis of variance. A *P* < 0.05 was considered significant. All data were analyzed using SPSS 19.0 software.

## 3. Results

### 3.1. Study Population

207 patients were diagnosed as ILD during the study period, and all were Han patients. 4 patients with environmental exposures and other known causes of ILD were excluded. 203 patients were included in our study. 63 patients met the criteria for CTD-ILD, 65 patients met the criteria for UCTD-ILD, and 75 patients met the criteria for IPF. The prevalence of CTD-ILD, UCTD-ILD, and IPF were 31.0%, 32.0%, and 36.9%.

### 3.2. Clinical Features

Clinical features of patients are shown in Table 2. The mean age of CTD-ILD was 57.24 ± 1.55 years, younger than patients with UCTD-ILD and those with IPF (*P* < 0.05). The percentage of male patients was 31.7% in the CTD-ILD group, significantly lower than the UCTD-ILD (63.1%) and IPF group (69.3%), and the percentage of ever smoker was significantly lower in CTD-ILD group than in the UCTD-ILD and IPF group, which indicate that young female ILD patients were more prone to be CTD-ILD patients, while older male ILD patients with smoking history were more prone to be UCTD-ILD and IPF patients. 

Cough, sputum production, dyspnea, and fatigue were common in all three groups but were less common in CTD-ILD group compared with CTD-ILD and IPF group. Symptoms of hemoptysis, chest discomfort, and chest discomfort were not seen in CTD-ILD patients. Symptoms suggestive of a connective tissue disease including arthralgia, dry eyes/dry mouth, Raynaud's phenomenon, proximal muscle weakness, and muscle pain were common in CTD-ILD patients and UCTD-ILD patients except proximal muscle weakness and muscle pain, which seemed to be specific to CTD-ILD groups. All of these symptoms were seldom seen in IPF patients. Face swelling and oral ulceration were only seen in few patients in the CTD-ILD group. The symptoms with significant difference between the CTD-ILD group and UCTD-ILD group are cough (*P* = 0.007), sputum production (*P* = 0.002), dyspnea (*P* = 0.018), chest discomfort (*P* = 0.004), chest pain (*P* = 0.001), proximal muscle weakness (*P* = 0.008), and muscle pain (*P* = 0.000). The symptoms with significant difference between the CTD-ILD group and IPF group are cough (*P* = 0.007), dyspnea (*P* = 0.11), chest discomfort (*P* = 0.000), chest pain (*P* = 0.011), skin rash (*P* = 0.000), arthralgia (*P* = 0.000), dry eyes/dry mouth (*P* = 0.043), Raynaud's phenomenon (*P* = 0.002), proximal muscle weakness (*P* = 0.004), and muscle pain (*P* = 0.000). The symptoms with significant difference between UCTD-ILD and IPF are cough (*P* = 0.000), skin rash (*P* = 0.000), arthralgia (*P* = 0.000), dry eyes/dry mouth (*P* = 0.012), and Raynaud's phenomenon (*P* = 0.001). These data suggest that ILD patients with symptoms of hemoptysis, chest discomfort, or chest pain were less likely to be CTD-ILD patients. ILD patients with symptoms suggestive of a connective tissue disease including arthralgia, dry eyes/dry mouth, Raynaud's phenomenon, proximal muscle weakness, and muscle pain were not likely to be IPF patients. Thus, a scan for evidence of CTD or UCTD is necessary in these patients.

### 3.3. Laboratory Findings

Serologic test results are shown in Tables 3 and 4. Autoantibodies were commonly seen in CTD-ILD groups; only ANA, RF, anti-SSB, and anti-Scl-70 were seen in UCTD-ILD patients, while only ANA and RF were found in IPF patients. 71% of CTD-ILD patients and 52% of UCTD-ILD patients had a positive ANA, higher than that of IPF (21%). Presence of positive RF was more common in CTD-ILD group than UCTD-ILD and IPF group, while there was no significant difference between UCTD-ILD and IPF group. Fifteen patients with CTD-ILD (24%) had one, 15 (24%) had two, and 22 (35%) had three or more abnormal serologic tests for autoantibodies. 35 patients with UCTD-ILD (54%) had one and 4 (6%) had two abnormal serologic tests for autoantibodies. Only 21 patients with IPF (28%) had one abnormal serologic test for autoantibodies. These data indicate that ANA and RF can be found in IPF patients, but if other autoantibodies were found in ILD patients, a diagnosis of CTD-ILD and UCTD-ILD should be considered.

Significant difference in other serologic tests between CTD-ILD groups and IPF group was seen in hemoglobin, platelet, ALT, AST, ALB, LDH, HBDH, IgM, C3, and C4. Significant difference was also found in ALB, LDH, and HBDH between CTD-ILD and UCTD-ILD group. Significant difference was only found in C3 between UCTD-ILD and IPF group.

### 3.4. HRCT Findings

HRCT image characteristics of these patients are shown in Table 5. All individuals showed UIP on HRCT scan. Almost all these patients showed irregular linear opacities in HRCT images. The most common images in all three groups were ground glass opacities, honeycombing, and consolidation. Subpleural curvilinear shadows were less common in all groups, 3% in CTD-ILD, 2% in UCTD-ILD, and 4% in IPF. The percentage of presence of consolidation in CTD-ILD patients was lower than UCTD-ILD patients (*P* = 0.034) and IPF patients (*P* = 0.023), while the presence of ground glass opacities in CTD-ILD patients was more common than UCTD-ILD patients (*P* = 0.002) and IPF patients (*P* = 0.006). However, there was no significant difference between UCTD-ILD group and IPF group in the image characteristics.

## 4. Discussion

Our study showed that patients with CTD-ILD, UCTD-ILD, and IPF were three distinct subgroups of diseases which differ from clinical features and serologic tests, and a systematic evaluation of symptoms and serologic tests in patients with ILD can identify these three subgroups. To date, this is the largest study to systematically evaluate patients with ILD to analyze the characteristics of CTD-ILD, UCTD-ILD, and IPF patients.

By retrospectively studied 203 cases of Chinese ILD patients, we found that CTD-ILD occupied about one-third of these patients. These results of our study were consistent with those reported by previous studies, with the prevalence of CTD-ILD varying from 12.4% to 34% [3, 7, 18, 19]. The UCTD-ILD as a nearly defined new group of ILD was also common in ILD patients, with prevalence of 32.0%, which was higher than CTD-ILD, but a little lower than IPF (36.9%). However, CTD-ILD patients and UCTD-ILD patients occupied about two-thirds of ILD patients, which meant that most ILD patients could be found to be autoimmune related; these patients may have a better prognosis, and immunomodulatory therapy should be considered.

We found that patients with CTD-ILD were more likely to be younger women and nonsmokers, with more antibody abnormalities and presentation of skin and muscle damage, as reported previously [9, 20, 21]. Symptoms of hemoptysis, chest discomfort, and chest pain were less likely to be presented in CTD-ILD patients. Patients with CTD-ILD also had lower levels of erythrocyte, hemoglobin, and hematocrit but highest levels of platelet and IgM in their serologic tests, but most of these parameters were within normal references. However, anemia was reported previously common in CTD, resulting from autoimmune hemolysis in most conditions [22–25]. 

IPF patients were more likely to be older male ILD patients with smoking history. ILD patients with symptoms suggestive of a connective tissue disease including arthralgia, dry eyes/dry mouth, Raynaud's phenomenon, proximal muscle weakness, and muscle pain were not likely to be IPF patients. Thus, a scan for evidence of CTD or UCTD is necessary in these patients. Only ANA and RF could be found in IPF patients, while other autoantibodies could be seldom found in these patients.

We also found that patients with UCTD-ILD were a distinct entity in patients with ILD, with their own clinical and serologic characteristics. The characteristics of UCTD-ILD patients seemed to lie between CTD-ILD and IPF, and the differentiation between UCTD-ILD and IPF appeared to be a little difficult. Unlike previous studies, patients with UCTD-ILD in our study were more likely to be men, and the mean age of them had no significance with that of IPF [6, 8]. Patients with UCTD-ILD had more extrapulmonary presentations and more antibody abnormalities than those of IPF. Skin rash, arthralgia, and Reynaud's phenomenon were common in UCTD-ILD [26], while not common in IPF. Autoantibodies of anti-SSB and anti-Scl-70 could be found in UCTD-ILD patients, while these two autoantibodies were not likely to be found in IPF patients. 

All individuals showed usual interstitial pneumonia on HRCT scan, and it was hard to distinguish IPF from CTD-ILD or UCTD-ILD based on HRCT scan itself. With no evidence for lung biopsy, a typical UIP pattern on HRCT scan did not exclude CTD-ILD or UCTD-ILD from IPF [6, 27]. Most characteristics of HRCT images of CTD-ILD were not different from those of other two groups, except for consolidation and ground glass opacities. Signs of consolidation were less common, and ground glass opacities were more commonly seen in HRCT images of CTD-ILD patients. However, images of consolidation and ground glass opacities were nonspecific, because even infection could display these features on HRCT scan [28]. There was no significant difference of these signs on HRCT scan between UCTD-ILD and IPF patients.

This study has the following limitations, Firstly, it is a retrospective study conducted in only one institute. Secondly, almost all of these patients refused to do lung biopsy for the possibility that lung biopsy is an invasive test and gives little contribution to treatment. 

## 5. Conclusion

CTD-ILD and UCTD-ILD patients occupied the most part of ILD patients, and patients with CTD-ILD, UCTD-ILD, and IPF differed in clinical features and laboratory findings. A systematic evaluation of symptoms and serologic tests in patients with ILD can identify CTD-ILD, UCTD-ILD, and IPF. In addition, there is much to be learned about the underlying pathogenesis of CTD-ILD and UCTD-ILD or IPF, and appropriate intervention trials should be conducted to learn about the treatment of these diseases.

## Figures and Tables

**Table 1:** Diagnostic criteria for patients with undifferentiated connective tissue disease (UCTD).

Diagnostic criteria	Presence of
Symptoms (at least one symptom)	(1) Skin rash
	(2) Arthralgia
	(3) Dry eyes/Dry mouth
	(4) Raynaud's phenomenon
	(5) proximal muscle weakness
	(6) Leg/foot swelling
	(7) Face swelling
	(8) Oral ulceration
	(9) Hand ulcers
	(10) Mouth ulcers
	(11) Raynaud's phenomenon
	(12) Morning stiffness
	(13) Recurrent unexplained fever

Serologic test (at least one test positive)	(1) Antinuclear antibody titer ≥1 : 160
	(2) Rheumatoid factor
	(3) Antidouble-stranded DNA
	(4) Anti-ribonucleoprotein antibody
	(5) Anti-Smith antibody
	(6) Anti-Sjoren syndrome A antibody
	(7) Anti-Sjoren syndrome B antibody
	(8) Anti-Scl-70
	(9) Anti-neutrophil cytoplasmic antibody
	(10) Anti-cyclic citrullinated peptide antibody
	(11) Anti-Jo-1
	(12) AKA

**Table 2:** Comparison of clinical characteristics among CTD-ILD, UCTD-ILD, and IPF patients.

	CTD-ILD	UCTD-ILD	IPF	P1	P2	P3
Subject (*n*)	63	65	75			
Age (years)	57.24 ± 1.55	63.58 ± 1.53	64.7 ± 1.68	<**0.05**	<**0.05**	0.614
Sex (M/F)	20/43	41/24	52/23	**0.000**	**0.000**	0.434
Ever smoker *n *(%**)**	15 (24)	35 (54)	35 (47)	**0.000**	**0.005**	0.397
Symptoms *n* (%)						
Cough	45 (71)	65 (100)	67 (89)	**0.000**	**0.007**	**0.007**
Sputum	33 (52)	51 (78)	48 (64)	**0.002**	0.167	0.061
Dyspnea	35 (56)	49 (75)	57 (76)	**0.018**	**0.011**	0.933
Fatigue	21 (33)	27 (42)	27 (36)	0.338	0.743	0.502
Hemoptysis	0 (0)	5 (8)	4 (5)	0.074	0.177	0.824
Chest discomfort	0 (0)	8 (12)	16 (21)	**0.004**	**0.000**	0.158
Chest pain	0 (0)	11 (17)	13 (17)	**0.001**	**0.001**	0.949
Skin rash	14 (22)	16 (25)	0 (0)	0.749	**0.000**	**0.000**
Arthralgia	19 (30)	14 (19)	1 (1)	0.265	**0.000**	**0.000**
Dry eyes/dry mouth	5 (8)	7 (11)	0 (0)	0.583	**0.043**	**0.012**
Raynaud's phenomenon	9 (14)	10 (15)	0 (0)	0.861	**0.002**	**0.001**
Proximal muscle weakness	8 (13)	0 (0)	0 (0)	**0.008**	**0.004**	—
Muscle pain	13 (21)	0 (0)	0 (0)	**0.000**	**0.000**	—
Recurrent unexplained fever	4 (6)	9 (14)	7 (9)	0.160	0.519	0.403
Leg/foot swelling	4 (6)	6 (9)	10 (13)	0.781	0.176	0.447
Face swelling	2 (3)	0 (0)	0 (0)	0.462	0.401	—
Oral ulceration	2 (3)	0 (0)	0 (0)	0.462	0.401	—

P1: Possibility when comparing CTD-ILD group and UCTD-ILD group. P2: Possibility when comparing CTD-ILD group and IPF group. P3: Possibility when comparing UCTD-ILD group and IPF group.

**Table 3:** Comparison of presence of autoantibodies among CTD-ILD, UCTD-ILD, and IPF patients.

	CTD-ILD	UCTD-ILD	IPF	P1	P2	P3
Subject (*n*)	63	65	75			
ANA *n* (%)	45 (71)	34 (52)	16 (21)	**0.026**	**0.000**	**0.000**
RF *n* (%)	26 (42)	9 (14)	7 (9)	**0.001**	**0.000**	**0.403**
Anti-ds-DNA *n* (%)	0	0	0	—	—	—
RNP *n* (%)	6 (10)	0	0	**0.011**	**0.006**	—
Anti-Smith *n* (%)	2 (3)	0	0	0.148	0.102	—
Anti-SSA *n* (%)	18 (28)	0	0	**0.000**	**0.000**	—
Anti-SSB *n* (%)	5 (8)	5 (8)	0	0.959	**0.011**	**0.014**
Anti-Scl-70 *n* (%)	4 (6)	4 (6)	0	0.964	**0.027**	**0.029**
ANCA *n* (%)	0	0	0	—	—	—
ACA *n* (%)	3 (5)	0	0	0.075	0.056	—
Anti-CCP *n* (%)	8 (13)	0	0	**0.003**	**0.001**	—
Anti-Jo-1 *n* (%)	2 (3)	0	0	0.148	0.120	—
AKA *n* (%)	6 (3)	0	0	**0.011**	**0.001**	—

P1: Possibility when comparing CTD-ILD group and UCTD-ILD group. P2: Possibility when comparing CTD-ILD group and IPF group. P3: Possibility when comparing UCTD-ILD group and IPF group.

**Table 4:** Comparison of laboratory findings among CTD-ILD, UCTD-ILD, and IPF patients.

	CTD-ILD	UCTD-ILD	IPF	P1	P2	P3
Subject (*n*)	63	65	75			
Erythrocyte (×10^12^/L, mean ± SEM)	4.07 ± 0.100	4.39 ± 0.100	4.41 ± 0.080	0.075	**0.028**	0.998
Hemoglobin (mg/L, mean ± SEM)	120.08 ± 3.186	130.31 ± 3.159	134.53 ± 2.244	0.071	**0.001**	0.645
Platelet (×10^9^/L, mean ± SEM)	207.84 ± 12.643	189.72 ± 10.102	168.69 ± 8.821	0.603	**0.037**	0.317
Hematokrit (1, mean ± SEM)	0.37 ± 0.011	0.40 ± 0.009	0.41 ± 0.007	0.129	**0.008**	0.695
Alanine aminotransferase ALT (IU/L, mean ± SEM)	38.58 ± 4.636	29.80 ± 4.871	25.96 ± 2.129	0.476	**0.045**	0.853
Aspartate aminotransferase AST (IU/L, mean ± SEM)	43.59 ± 4.500	30.43 ± 3.671	27.25 ± 1.630	0.074	**0.003**	0.815
Serum albumin ALB (mg/L, mean ± SEM)	32.33 ± 0.741	35.33 ± 0.571	36.75 ± 0.738	**0.005**	**0.000**	0.272
Lactate dehydrogenase LDH (IU/L, mean ± SEM)	331.81 ± 18.282	256.10 ± 12.395	260.56 ± 15.083	**0.003**	**0.010**	0.994
Hydroxybutyric dehydrogenase HBDH (IU/L, mean ± SEM)	273.28 ± 16.568	210.62 ± 11.161	215.16 ± 13.132	**0.007**	**0.021**	0.991
Immunoglobulin G (g/L, mean ± SEM)	15.57 ± 1.046	14.36 ± 0.493	13.10 ± 0.646	0.655	0.137	0.311
Immunoglobulin A (mg/dL, mean ± SEM)	2847.26 ± 187.573	3051.06 ± 192.602	2781.23 ± 204.235	0.834	0.993	0.710
Immunoglobulin M (mg/dL, mean ± SEM)	1671.00 ± 152.988	1462.94 ± 202.364	1110.78 ± 73.626	0.799	**0.004**	0.285
Immunoglobulin E (mg/dL, mean ± SEM)	189.19 ± 44.544	166.33 ± 31.581	183.73 ± 47.300	0.966	1.000	0.986
C3 (g/L, mean ± SEM)	0.85 ± 0.033	0.92 ± 0.024	1.01 ± 0.026	0.306	**0.001**	**0.034**
C4 (g/L, mean ± SEM)	0.18 ± 0.010	0.33 ± 0.140	0.21 ± 0.008	0.624	**0.022**	0.785

P1: Possibility when comparing CTD-ILD group and UCTD-ILD group. P2: Possibility when comparing CTD-ILD group and IPF group. P3: Possibility when comparing UCTD-ILD group and IPF group.

**Table 5:** HRCT findings of ILD patients.

	CTD-ILD	UCTD-ILD	IPF	P1	P2	P3
Subject (*n*)	63	65	75			
Consolidation *n* (%)	11 (17)	22 (34)	26 (35)	**0.034**	**0.023**	0.919
Ground glass opacities *n* (%)	42 (67)	34 (52)	42 (56)	**0.002**	**0.006**	0.662
Irregular linear opacities *n* (%)	59 (94)	62 (95)	71 (95)	0.666	1.000	1.000
Traction bronchiectasis *n* (%)	6 (10)	11 (17)	13 (17)	0.217	0.185	0.949
Honeycombing *n* (%)	28 (44)	32 (49)	31 (41)	0.587	0.713	0.349
Subpleural curvilinear shadows *n* (%)	2 (3)	1 (2)	3 (4)	0.716	0.740	1.000

P1: Possibility when comparing CTD-ILD group and UCTD-ILD group. P2: Possibility when comparing CTD-ILD group and IPF group. P3: Possibility when comparing UCTD-ILD group and IPF group.
